# Review on Portable-Powered Lower Limb Exoskeletons

**DOI:** 10.3390/s24248090

**Published:** 2024-12-18

**Authors:** Chunyu Jiang, Junlong Xiao, Haochen Wei, Michael Yu Wang, Chao Chen

**Affiliations:** Department of Mechanical and Aerospace Engineering, Monash University, Clayton, VIC 3800, Australia; chunyu.jiang@monash.edu (C.J.); junlong.xiao@monash.edu (J.X.); haochen.wei@monash.edu (H.W.); mywang@gbu.edu.cn (M.Y.W.)

**Keywords:** lower limb exoskeleton, design, control strategy, sensors, fusion

## Abstract

Advancements in science and technology have driven the growing use of robots in daily life, with Portable-Powered Lower Limb Exoskeletons (PPLLEs) emerging as a key innovation. The selection of mechanisms, control strategies, and sensors directly influences the overall performance of the exoskeletons, making it a crucial consideration for research and development. This review examines the current state of PPLLE research, focusing on the aspects of mechanisms, control strategies, and sensors. We discuss the current research status of various technologies, their technological compatibility, and respective benefits comprehensively. Key findings highlight effective designs and strategies, as well as future challenges and opportunities. Finally, we summarize the overall status of PPLLE research and attempt to shed light on the future potential directions of research and development.

## 1. Introduction

The energy consumption of the legs ranks first among human organs and is crucial for human daily activities. Nowadays, PPLLEs are commonly divided into three broad categories, namely augmentation exoskeletons, assistive exoskeletons, and therapeutic exoskeletons, despite there being some devices that cross over between the latter two categories [1]. Augmentation exoskeletons [2,3] enhance the physical capabilities of healthy individuals, such as mobility, agility, and load capacity. Assistive exoskeletons [4,5,6,7,8] are designed to assist individuals with functional disabilities, aiding in coordinated exercises and rehabilitation to restore their exercise capacity. With the guidance of physiotherapists, the therapeutic exoskeletons can provide force or torque according to certain patients. Specifically, exoskeletons for elderly people can detect abnormal gait to avoid falling [9,10]. Since this review focuses on portable devices, large rehabilitation exoskeletons designed for patients with severe disabilities like paraplegia are out of scope.

Generally, Powered Exoskeletons (PEs) can achieve superior performance compared to Unpowered Exoskeletons (UEs), enabling users to complete tasks that were previously unattainable. PEs utilize external energy sources to generate mechanical power, surpassing the capabilities of UEs, which are primarily designed to enhance ergonomics with limited energy storage. Devices designed for augmentation and assistive purposes offer enhanced flexibility and mobility in daily life due to their portable design. These portable devices are more affordable and can be easily integrated into various activities. These portable devices are more readily affordable and can be easily integrated into various activities. For example, the commercial gait assists exoskeleton devices [6] and provides powered hip and knee motion, which enable patients to stand and walk. Recently, it has just been approved by the Food and Drug Administration (FDA) in the United States, extending the function to walking up stairs and over curbs.

However, the increasing complexity of the Human–Robot Interaction (HRI) poses significant challenges in the design and control of active exoskeletons. Therefore, the objective of this review is to provide a comprehensive overview of the current advancements in PPLLEs and explore avenues for further progress in the field. This paper is divided into three parts. Firstly, the mechanism of PPLLEs was divided into rigid, flexible, and soft based on flexibility. We analyzed the relevant performance data and conducted a comprehensive comparison. By evaluating the advantages and disadvantages of each type, we discuss the specific scenarios where they excel. Secondly, control strategies are classified into decision-level and execution-level categories. We introduced emerging technologies and evaluated strategy incorporation approaches that overcome the limited performance of individual ones. Thirdly, the different roles of different sensors in the control algorithm will be summarized and the trends in sensor fusion will be analyzed. Last but not least, the advantages of converged technology and the current research challenges will be discussed with possible innovative solutions.

## 2. Mechanism Design

The mechanism of PPLLEs is classified into three main types based on flexibility: rigid, soft, and flexible. Rigid PPLLEs utilize rigid actuators and links; soft PPLLEs employ flexible actuators and links; and flexible PPLLEs combine both approaches, integrating rigid actuators with flexible links to achieve a balance between rigidity and versatility. The development trends of these PPLLE types, along with their respective merits and limitations, are outlined as follows.

### 2.1. Rigid PPLLEs

Hydraulic actuators are widely utilized for controlling and maneuvering heavy objects due to their high force output. Their large force output enables easy support of the wearer’s body, making them a preferred choice for PPLLEs. They are preferred for tasks such as load-bearing and assembly line operations, especially in environments with stable energy sources. One can conclude that hydraulic actuators are more commonly used to assist the hip and knee rather than the ankle. This preference arises because ankle joints require minimal operating torque and the natural flexibility characteristic of the ankle that can adjust to varying terrains because of tendons and gravity. In recent years, Maowen et al. [11,12] developed a PPLLE for hip and knee assistance with a maximum thrust of 1770 N and a total weight of 10 kg. Compared to the previous prototype, the weight of the hydraulic actuator was reduced by approximately 40%, while the power density increased by nearly 1.6 times. Similarly, Rituraj et al. [13] prioritized lightweight design and designed a knee hydraulic PPLLE that weighs only 2.8 kg per leg and delivers a maximum torque of 100 Nm.

Motors are also commonly used in the field of PPLLEs. The application of motors in exoskeletons can be attributed to their various advantages. Firstly, motors are compact and can be designed to cooperate well with the human body, sharing similar rotating motion with human joints. Secondly, the motor’s range of motion is limited only by the rigid structure around it. This advantage makes it easy to have a larger range of motion. Thirdly, motors can achieve high torque outputs through the use of transmission mechanisms, which can scale up torque levels with transmission ratios ranging from one to several hundred. Compared with hydraulic drives, motor drives are beginning to be used for ankle assistance due to their small size. The presence of encoders allows for more precise control. Additionally, the weight can be further reduced through careful structural and transmission system design. For instance, based on a custom quasi-direct drive actuation consisting of a DC motor unit and a transmission mechanism with three gears, the knee exoskeleton developed by Long et al. [14] is notably compact, weighing only 1.3 kg per leg, with an additional 0.65 kg for the external energy source.

Research on hydraulic and motor-actuated rigid PPLLEs has been declining in recent years. While rigid structures offer significant reliability for supporting the bodies of disabled individuals and can provide the high force and torque needed for industrial tasks, they face limitations in HRI. The research aims to address the misalignment issues in rigid knee PPLLEs. Approaches such as using linear guide rotary joints and sliders for self-alignment have been explored [15,16]. Additionally, Marco et al. [17] proposed a general method for design and analysis to enhance their practical applications. However, these solutions introduce increased design complexity compared to flexible and soft exoskeletons, which inherently possess passive self-aligning capabilities. Customization requirements and challenges related to comfort and safety have hindered the widespread adoption of this technology.

Flexible PPLLEs have become the mainstream alternative [18], as they combine the benefits of both softness and rigidity. They use a hydraulic actuator or motor with soft connecting components—such as springs, cables, and variable stiffness mechanisms. These technologies enhance user comfort, safety, and performance, making flexible PPLLEs more suitable for broader applications.

### 2.2. Flexible PPLLEs

#### 2.2.1. SEA

Linear, nonlinear, and spiral springs are commonly used as SEAs in PPLLE due to their advantages, including high-fidelity torque control, reduced power requirements, enhanced safety and robustness, lower mechanical impedance, and shock tolerance [19]. For example, Qing et al. [20,21] proposed a novel nonlinear Series Elastic Actuator (nSEA), which could significantly reduce mechanical impedance and improve back drivability. It can be used in hip joints to address actuation transparency, safety, and other existing SEA problems, for instance, significantly reducing the root-mean-square (RMS) error of assistive torque from 1.571 Nm to 0.238 Nm. Similarly, the compact exoskeleton joint module [22] incorporates an nSEA, utilizing a unique crank slider mechanism and a set of linear springs for nonlinear stiffness (ranging from 220 to 440 Nm/rad) of its physical impedance, thereby improving the torque effect.

RSEA is the other kind of SEA, which is more compact and can be directly assembled in joints and connected with motors. They lower mechanical impedance, resist impact loads, and improve system energy efficiency. The PPLLE proposed by Meijneke et al. [23] used a modular approach that allows the reconfiguration of PPLLE with eight powered SEA joints ultimately. Under the concept of stiffness compensation-based gait assistance [24,25], the series-wedge structure was designed to enhance comfort during HRI and adapt passively to the user’s body curvature without hindering movement. Zhang et al. [26] developed a PPLLE using series-wedge structures, achieving energy-efficient walking and running and reducing the metabolic rate by 10.9% for walking and 6.2% for running. The HRI problem is considered from the structural and algorithmic perspectives to improve the torque tracking performance [27]. All of the related research data on SEA are summarized in Table 1.

As discussed, springs are commonly used in PPLLEs, effectively combined with traditional rigid actuators (hydraulic and motors) and emerging structures like variable stiffness to reduce mechanical impedance and increase safety, stability, and energy efficiency. Depending on the application, springs or similar mechanisms can significantly improve exoskeleton performance.

#### 2.2.2. Variable Stiffness

To mimic the efficient passive behavior of the joint and provide active assistance in locomotion, the concept of a Variable Stiffness Actuator (VSA) PPLLE has been proposed. It behaves similarly to biological muscles and can adapt to multiple applications to actively change the stiffness of the exoskeleton joint, which is critically important in keeping wearers safe, improving the performance of shock absorption, and reducing the complexity of control and energy consumption [28].

There are two main types of VSAs. The first is brake structure, such as magnetorheological brake structure [29] and bicycle brake similar structure [30]. They can stop and release the rotary movement in a short time. Recently, researchers have increasingly focused on the other types of VSAs, which aim to achieve continuous control of stiffness, rather than just discrete control (either 0 or 1). For example, in 2021, Kimura et al. [31] used a pneumatic artificial muscle and a pull spring to achieve variable stiffness (0 to 0.197 Nm/deg); the experiment confirms that its behaviors are similar to the theoretical one-sided spring antagonized joint. In the same year, a load-adaptive actuator [32] was proposed for actuating ankle exoskeletons through an inverted slider-crank mechanism. When the motor drives the ball screw to rotate, the leaf spring will open and its stiffness will increase. A similar mechanism has been used for MeRIA [33].

Springs can also be used for joints directly connected to rotating mechanisms to achieve a large range of rotation and variable stiffness. Sarani et al. [34] designed a prototype using this principle to improve the capacity to store energy and change the stiffness of joints. The range of motion reaches ±180°, and the range of stiffness reaches 98 to 533.6 N m/rad. Zhu et al. proposed the RVSA [35], which can achieve smaller, medium, and larger stiffness ranges through reconstruction (changing the winding method of the wire rope). It makes the structure more compact and reasonable and improves the energy storage capacity of the actuator. Hu et al.’s exoskeleton [36] combines variable stiffness with the energy storage capability of springs. It was found that, under optimal stiffness assistance, muscle fatigue in the rectus femoris was reduced by 8.5%. Table 2 provides the related research data of VSA. Although there are several advantages of VSA, the problems of VSA are also obvious. The system is relatively bulky and lacks robustness. Also, the multiple components and complex structure require great effort in design and manufacturing.

#### 2.2.3. Cable

Most existing PPLLEs are heavy and the weights are concentrated on the limbs, which can cause unnecessary inertial vibration. PPLLE driven by cables (PPLLEC) can overcome these problems by fixing the drivers to the waist or incorporating them into backpacks, enabling remote driving. This driving method can enhance safety during HRI and greatly reduce the load on the legs and the discomfort of the wearer [37].

As shown in Table 3, a Variable Radius Drum-cable (VRD) [38] mechanism with a variable transmission ratio is employed to reduce the effect of the harvester on the users’ gaits. Compared with the other harvesters that mainly harvest during swing extension, this harvester can store energy during swing and stance flexion where a large amount of negative work is available. COWALK-Mobile 2 [39] consists of two driving pulleys, one driven pulley, and a connecting link. Two driving pulleys are connected to the driven pulley in different directions to achieve a bidirectional rotary. The cable-differential mechanism makes it possible to change the required torques and speeds of the actuators by changing the radius of the pulleys. Kieuvongngam et al. [40] proposed a novel PPLLEC using two cables wrapped in opposite directions for bidirectional rotation. Their experiment demonstrated a more than 73% reduction in average robotic assistance torque. Orekhov et al. [41] developed an ankle exoskeleton with a parallel elastic element that stored and returned energy in parallel to a cable-driven ankle joint during the stance phase, reducing peak motor current by 14–20%. The peak current required by the motor is reduced by 14–20%. Chen et al. [42] proposed a switchable ankle-assistance exoskeleton that can alternate between single- and dual-motor ones to adapt to different users and tasks. A structure proposed by Zhong et al. [43] combines SEA and the cable to improve high-force fidelity, and the weight of one leg is only 0.7 kg.

Despite these benefits, the optimal number of cables and cable configurations to achieve the best performance remains elusive [44]. Furthermore, the Bowden cable may experience intermittent instantaneous relaxation, which has high requirements on the control system. The actuator’s stiffness and the volume of the motor are large when high power output is required. In addition to the research discussed above, cables are currently used more widely in soft exosuit, which performs better in certain aspects, and detailed information will be provided later.

### 2.3. Soft Exosuit PPLLEs

The concept of soft exosuits as fully soft wearable devices has received high attention from researchers in recent years. Compared with rigid exoskeletons and flexible exoskeletons, soft exosuits have several advantages [45]: they can be very light and have extremely low inertia; they are low profile and can be worn underneath regular clothing; and they are more comfortable and secure than other structures. A lot of studies have focused on soft exosuits driven by cables and pneumatics.

#### 2.3.1. Portable Lower Limb-Powered Exoskeleton-Cable Exosuits (PPLLEC-EXOSUITs)

As shown in Table 4, PPLLEC-EXOSUITs have been extensively studied because of their rapid response, lightweight nature, and simple structure. Each design serves a specific purpose. For example, a swimsuit, an underwater lower-extremity soft exoskeleton that could assist with swimming motions, has been designed by Wang et al. [46], where the assistive force was applied to the bottom of the fins via soft cables. In 2021, inspired by time division multiplexing and the symmetry of walking motion, Ye et al. [47] also used only one motor during a gait cycle to assist hip-plantar flexion of both legs in different phases. Similarly, Ma et al. [48] relied on springs and two cables to enable one motor to control knee extension and ankle plantarflexion for each leg. A new structure was proposed that incorporates SEA into the system [49,50] to reduce muscle activity during stair ascent and descent. The focus was on decreasing the required tension by using a lightweight knee extension, as high cable forces are needed and the small natural moment arm of the knee extensors cannot meet these demands. Inspired by the flexor and extensor of biological joints, AADLDS [51] was proposed for removing misalignments between biological joints and artificial joints. A design that enables hip motion in all DoFs can achieve versatile gaits [52]. A lightweight exoskeleton incorporating a unidirectional ankle-knee gait clutch was proposed, which allows for the sequential provision of auxiliary torque to the knee and ankle joints during the stance phase of gait [53]. Xu et al. [54] propose a bioinspired cable-driven actuation system capable of providing anisometric contraction assistance or nearly acting as a transparent device in an efficient manner, which could be directly applied to existing cable-driven wearable robots. Their design could reduce soleus muscle activity by 27.32% compared with normal walking.

#### 2.3.2. Portable Lower Limb-Powered Exoskeleton-Pneumatic Exosuits (PPLLEP-EXOSUITs)

Similar to PPLLEC-EXOSUIT, PPLLEP-EXOSUIT is also capable of addressing heavy-weight challenges due to the remote drive capability of the air pump. However, PPLLEP-EXOSUITs excel in terms of enhancing the robustness and compliance of the structure. They typically utilize soft materials to achieve safer and more reliable actuation, avoiding the need for springs or other complex mechanisms. As a result, PPLLEP-EXOSUIT designs commonly have a lighter weight. In 2020, soft airbags as actuators have been used in the ankle joint [55], which is lightweight and form-fitting for the user and can be easily worn over a variety of athletic shoes of various sizes, including in the knee joint [56] during sit-to-stand for realizing stable and comfortable torque output. To increase the response speed of an inflatable actuator, Park et al. [57] proposed an inflatable wrinkle actuator with fast inflation and deflation responses. The experiment proved that the inflation and deflation times were 0.40 s and 0.16 s, respectively. In 2022, a SWAG [58] for hip flexion rehabilitation has been designed to realize lightweight, flexible, and low-cost. This rotary actuator can reduce muscle activity by 43.5%. To enable the rapid and scalable mass fabrication of robust pouch motors, Yilmaz et al. introduced a novel textile-based pneumatic actuator [59]. This innovation utilizes computerized knitting technology, incorporating ultrahigh molecular weight polyethylene yarn (Spectra) and conductive silver yarns.

As shown in Table 5, PPLLEP-EXOSUITs’ structures are very simple but can also perform high performances, which makes it another mainstream actuator method in exoskeletons. From the development of pneumatic exosuits in recent years, we can see that as long as better new soft actuator structures and materials appear, they can be well used in exoskeletons, and pneumatics are very light and soft, which is very suitable for this HRI scenario. However, the service life of the material and the drive speed are still a challenge.

#### 2.3.3. Other PPLLEs

Polyvinyl chloride (PVC) has been used in several fields due to its high power-to-weight ratio and fast response speed. In the aspect of PPLLE, Maeda et al. [60,61] proposed variable stiffness spats actuated by PVC gel, which can assist walking by restraining and releasing body motion with different levels of stiffness. The PVC spats are arranged in an X shape. When the power is on, they will contact and bring the knee joint up. When the power is off, it will recover to the initial condition. The authors also designed multi-layer PVC gel artificial muscles (PGAMs) [62,63,64] for hip joint assistance using a stainless mesh electrode as the anode to enlarge the deformation and stainless foil as the cathode. When the DC field is applied, contraction deformation occurs because the gels move into the holes of the mesh. Similarly, when the DC field is off, the structure returns to its initial condition because of the material elasticity of PVC gels. Shape memory alloys (SMAs) have also been used in this field due to their similar properties. For example, SMA wires have been used in ankle assistance [65]. It can contract within 0.5 s under 0.8 A electrical current and within 0.2 s with 1 A electric current. Dielectric Elastomer Actuators (DEAs) can also be used for ankle assist [66], with properties similar to PVC.

Using PVC, SMA, or DEA as actuators in PPLLE offers several benefits, including a high power-to-weight ratio and the avoidance of complex systems and structures such as air pumps and rigid supports. However, PVC and DEA require a high voltage to drive and need careful consideration when it comes to the safety of HRI. SMA produces high heat when actuated and needs time to cool down. Additionally, SMA actuators have a finite lifespan due to the fatigue properties of the material. Over time, repeated actuation and cooling cycles can cause degradation and eventual failure of the SMA actuator. We can see that there are not many studies in this area, mainly because there are still many problems to be solved, such as safety, reliability, service life, control accuracy, etc. However, it is undeniable that with the development of emerging soft robots and actuators, this field will be more and more favored by researchers. After all, there is still a big gap in this research field.

## 3. Control Strategy

**Figure 1:**
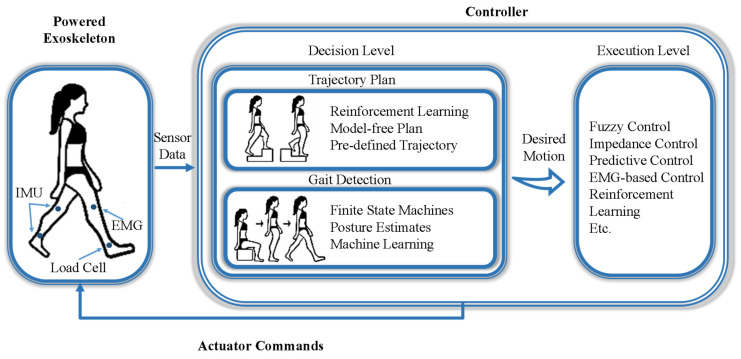
The exoskeleton control flow chart. First, the sensors collect physical and physiological data. Secondly, the received data were used to determine the user’s motion intention and plan the corresponding trajectory. Then, the activity signal was transmitted to the actuator for accurate motion execution.

Currently, a universal control strategy has not yet emerged. The trend is to incorporate two or more control strategies and adaptively change system parameters to enhance overall performance and functionality. To ensure a safe and harmonious HRI, it is significant to devise an accurate and smooth control system that can accurately interpret and follow the wearer’s intentions. According to this purpose, as shown in Figure 1, the exoskeleton control framework can be divided into two levels:

(a) Decision level: At this level, the exoskeleton incorporates the user’s body posture and consciousness, determines precise motion instructions that correspond to the wearer’s desired actions, and generates/quotes the corresponding/preloaded standardized locomotion trajectories such as walking, standing, sitting, and ascending stairs;

(b) Execution level: The motion instructions obtained from the decision level are implemented by the exoskeleton’s actuators to achieve accuracy and precision in desired movements.

### 3.1. Decision Level

#### 3.1.1. Gait Detection

Gait detection discerns the current actions of a user during locomotion and predicts their future intentions. It can determine whether the user is sitting, walking, climbing, or engaged in other activities, thereby providing a suitable build-in program and predicting the next steps. This process involves analyzing various physical and physiological parameters with refined specific threshold values.

(a) Rule-based classifier

Human locomotion is commonly classified into distinct motion subsets that correlate with the individual’s neural activities and physical positioning. To achieve this, sensors such as Inertial Measurement Units IMUs, EMGs, and gyroscopes are employed to capture the user’s present state data. Once the acquired data satisfy predefined trigger conditions, a classifier is immediately activated, facilitating the transition from one gait to another. Finite State Machines (FSMs), such as the one utilized in MINDWALKER, are commonly employed for this purpose. It utilized FSM to imply corresponding assistant walking states if the Centre of Mass ground projection falls in the desired quadrant, which is estimated by IMU [67].

Additionally, posture estimation can be utilized to adjust the gait pattern based on the pilot’s intention, which can be expressed through arm movements or unconstrained parts of the current joint and foot setup. This approach allows the pilot to communicate their desired motion intent through specific postures, enabling the exoskeleton to adapt accordingly.

(b) Pre-trained classifier

Machine learning (ML) techniques, including Naive Bayes (NB), Support Vector Machine (SVM), Gaussian Process (GP), Deep Learning (DL), Reinforcement Learning (RL), etc., with their superiority, offer remarkable capabilities in learning targets behaviors and accurately predicting outcomes based on past observation data. Exoskeletons are typical examples of multi-input-multi-output (MIMO) systems, making the mapping of sensor readings to the accurate control complex and nonlinear. Compared to rule-based classifiers, pre-trained classifiers are more efficient in extracting valuable information from the wearer’s physical state and biological signals, especially when dealing with contaminated raw data with measurement, environmental, and process noise since they are dealing with noise. Hence, by learning from a large dataset categorized into different modes, ML classifiers automatically generate decision criteria that consider similarities in movement performance. Park et al. [68] developed an online sparse Gaussian Process regression algorithm to learn human motion intent using the physical Human–Robot Interaction (pHRI) and knee joint torque, which achieves a good trade-off between system performance and computation cost. Moreover, Kang et al. [69] proposed a sensor fusion-based neural network model to estimate the gait phase for speeds ranging from 0.6 to 1.1 m/s.

#### 3.1.2. Trajectory Plan

The PPLLE is designed to provide assistance to users under specific conditions. Traditionally, most PPLLEs are programmed with predetermined trajectories to assist users. However, there are new approaches emerging that enable real-time planning.

(a) Pre-defined trajectory

To achieve the desired gait control in exoskeletons, one approach is to develop a mathematical model that captures the system’s physical characteristics. This involves modeling the exoskeleton theoretically, considering its dynamics, kinematics, and mechanical properties. Moreover, clinical gait analysis and normal human gait data are valuable references for generating the model; Prakash et al. [70] provide a comprehensive survey of free available gait datasets. However, due to variations in human characteristics, various types of sensor noise, and potential inaccuracies in mathematical models, it can be challenging for the proposed method to perfectly align with every individual user.

(b) Model-free trajectory

The wearable exoskeleton faces a situation with strong coupling, high nonlinearity, uncertainty, and disturbance. Consequently, developing an accurate mathematical model for such a system is challenging. In light of this, model-free control emerges as an alternative technique, reducing the need for detailed dynamics information.

Compared with a predefined trajectory, a model-free controller using an ultra-local model that only considers the applied control input, lumped uncertainty, and system output, ignoring the physical state of the embedded subject, can formulate a simplified dynamic relation for complex systems. The ultra-local model always incorporates intelligent PD (iPD) controllers, which can adjust parameters to match the model’s performance. However, to enhance the tracking precision and disturbance observation, Han et al. [71] applied the time-delay estimation (TDE) technique and sliding model control; Zhang et al. [72] used TDE and Radial Basis Function (RBF) neural network; Han et al. [73] used a Sigmoid function-based Tracking Differentiator (STD) and linear discrete-time extended state observer (LDESO); and Sun et al. [74] used a uniform robust exact disturbance observer (UREDO) and nonsingular terminal sliding mode control. Simulation and experimental results verified the effectiveness of the proposed method, specifically in better angle tracking performance.

Reinforcement learning offers an alternative approach to realizing self-planned trajectories in exoskeleton systems. The principle behind RL can be described as follows: it is a process of deciding what to perform under a set of alternative responses to maximize a reward determined by the supervisor. Under a reasonable and effective reward setting, the RL algorithm automatically seeks long-term and maximum overall reward to achieve an optimal solution. By leveraging this reward-driven framework, the exoskeleton can iteratively improve its performance over time, continuously refining its trajectory to achieve better outcomes. This approach eliminates the need for pre-defined trajectories and enables the system to dynamically adjust its actions to maximize rewards or achieve specific objectives [75,76,77,78].

### 3.2. Execution Level

**Figure 2:**
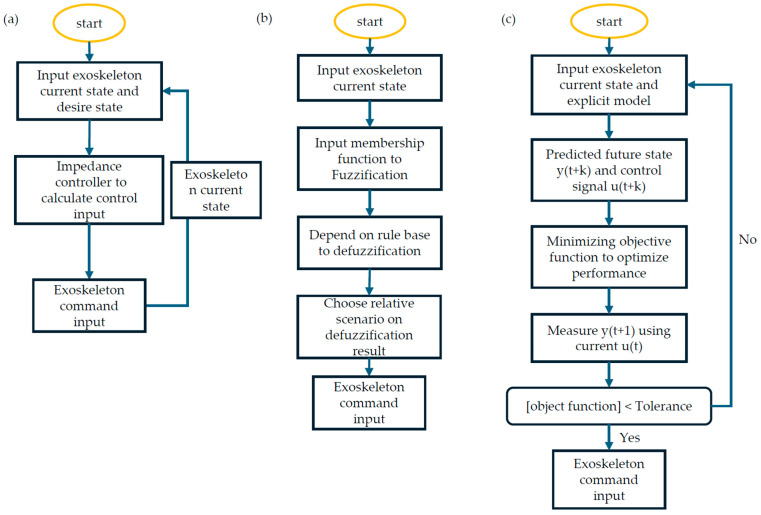
Flowchart of different control strategies. (**a**) for impedance control, (**b**) for fuzzy control, and (**c**) for predictive control.

The execution level refers to the signal given by the upper level and applied directly to the exoskeleton to achieve the desired locomotion. However, generating precise objectives can be challenging due to inevitable inherent errors and potential actuator failures. Compared with the traditional PID method, which is inefficient in handling complex dynamic environments, state-of-the-art control strategies are exclusively or collectively implemented to automatically compensate for variations in system dynamics. This ensures that the system remains robust and stable. Figure 2 is a flowchart discussing different control strategies.

#### 3.2.1. Impedance Control

The primary advantage of impedance control is that the exoskeleton can dynamically adjust its response by actively modulating its impedance based on the encountered forces. It remains inactive unless the subject’s motion diverges from the anticipated trajectory of movement. This feature enables the exoskeleton to maintain the desired optimal motion trajectory effectively since the human impedance shows nonlinear behavior [79,80]. For example, Huo et al. [81] presented a nonlinear impedance reduction control (IRC) for a lower limb knee assistive device, which provided active impedance assistance. The effectiveness of the proposed methods was evaluated by measuring the root mean square of the EMG signals, which reflect the level of human torque development. Their experiment results show promising achievements, with a reduction of approximately 44.7% in the original impedance. Similarly, Karavas et al. [82] proposed a tele-impedance-based assistive control scheme for a knee exoskeleton device, which developed an EMG-based musculoskeletal model calibrated with different biomechanical studies to map reference stiffness input for the skeleton. The experiment results demonstrated that the proposed control scheme could provide about 30% of the required standing-up torque. Additionally, Yaohui et al. [83] present an event-triggered impedance control, which transfers the control problem to an optimal problem. To trigger the optimal controller, a critical neural network is developed through the framework of reinforcement learning with a modified gradient descent method.

#### 3.2.2. Predictive Control

Compared with conventional control methods that control the current state to achieve precise control, Model Predictive Control (MPC) obtained control action by solving, at each sampling instant, a finite horizon optimal control problem where the initial state is the current state of the system. The optimization process produces a finite control sequence, but only the first control action is applied to the plant. Dos Santos et al. [84] proposed an MPC method to determine optimal stiffness parameters for exoskeletons designed for rehabilitation purposes. To maximize the effectiveness of the rehabilitation process, the torque provided by the robot should guarantee the performance of normal gait and promote the active participation of the patient. The experimental results showed a positive impact of estimating patients’ torque compared to classical methods. Moreover, Christopher et al. [85] introduced an MPC that can provide optimal assist-as-needed control by integrated fuzzy logic to map key modes and EMG signals to predict trajectory following accuracy.

#### 3.2.3. EMG-Based Control

Electromyography (EMG) is a crucial biological signal that directly reflects human muscle activities, making it suitable for estimating force with a reasonable degree of accuracy as it is generated during muscle contraction. However, some challenges need to be addressed, including blended motion signals, variable individual activity levels, and multiple motion-muscle mapping. Gui et al. [86] present an adaptive EMG-based method for estimating active joint torque to improve the effectiveness of rehabilitation training. With radial basis function neural networks (RBFNNs), the controller eliminated the need for calibration in the EMG-torque model. Although the study demonstrated the positive performance of the proposed method, the proposed method was limited under the stance phase because of unknown ground reaction forces. Moreover, Gandolla et al. [87] recorded EMG activity from the lower limb muscles to identify the most effective trigger point for Ekso. Moreover, in order to enhance the transparency of the knee joint in an exoskeleton, Chen et al. [88] implemented a novel energy kernel method to estimate the activation level of muscle, combined with position and interactive force to design an online adaptive predicting controller. Moreover, Ao et al. [89] proposed an EMG-driven Hill-type Neuromusculoskeletal Model (HNM), which can estimate ankle joint torque for better ankle tracking accuracy. Zhao et al. [90] used an EMG signal to predict both motion intention and joint impedance without adding a calibration process.

#### 3.2.4. Fuzzy Control

Fuzzy logic control (FLC) is a highly valuable control theory for addressing unstructured uncertainties and multi-input-multi-output (MIMO) nonlinear systems. The effectiveness has been proven in industrial applications, such as air conditioners to adjust their actions to minimize errors in both temperature and humidity. In realistic scenarios involving exoskeletons, it is difficult to structure an accurate model because of dynamic uncertainties, disturbances, and individual variations. Therefore, fuzzy control, with its robustness, simplicity, model-free nature, and efficiency, is implemented as both the underlying core control and in combination with other methods in order to address these challenges.

For example, Yin et al. [91] proposed an experience-based fuzzy rule to update the proper system parameters with the support of a muscle-tendon complex model. For the core set of the system, fuzzy control rule bases are generated using prior knowledge from either human expertise or labeled training data. In addition, Li et al. [92] proposed an innovative adaptive fuzzy control method that eliminates the built-in torque-sensing unit. This control approach utilized fuzzy logic to approximate the unknown continuous functions while employing disturbance observers to capture various sources of uncertainty, including viscous friction, gravity, payloads, etc., as one unknown term. Extensive experiments were conducted to compare position tracing accuracy with the PD controller and the results showed a significant reduction in tracking errors. Additionally, Huang et al. [93] developed a fuzzy enhanced adaptive admittance control strategy for a wearable walking exoskeleton that can reshape step trajectory over consecutive gait cycles. It changes the predetermined reference trajectory under the wearer’s walking intention through the HRI force. Moreover, Sharma et al. [94] designed an efficient fuzzy logic PID (FLC-PID) control with a nature-inspired DragonFly Algorithm (DFA) for obtaining the control parameters. In principle, the FLC-PID controller is divided into FLC-PI and FLC-PD controllers, which reduce system complexity and processing time, to flexibly change three gains, proportional, integral, and derivative, for better adaptability with only two inputs, error and rate of change in error. Moreover, Narayan et al. [95] used the bonded linear quadratic regulator, which aimed to linearize nonlinear input-output relations and the adaptive neuro-fuzzy inference system (ANFIS) to balance the effect of payload uncertainties and external disturbances during passive-assist gait training for a lower limb exoskeleton system. Sun et al. proposed a reduced adaptive fuzzy decoupling control system for a lower limb exoskeleton rehabilitation system to solve the uncertain nonlinear MIMO system, decouple the entire MIMO system into several Multi-Input-Sulti-Output (MISO) subsystems, and merge with a reduced adaptive fuzzy system to reduce possible chattering phenomena for control stability.

#### 3.2.5. Reinforcement Learning/Deep Reinforcement Learning Control

Reinforcement learning (RL) is a powerful solution already, but as a derivative of reinforcement learning, deep reinforcement learning (DRL) is more efficient. In RL, the reward of each state and action needs to be stored and analyzed by the controller to pick the best action that can maximize rewards. However, when the environment becomes complicated and the required storage space becomes huge, it is no longer feasible to store all information. So, a neural network is created to predict the reward for a certain input.

Huang et al. [75] present an adaptive control strategy that uses dynamic movement primitives (DMPs) to model motion trajectories and reinforcement learning to optimize the sensitivity factors of the controller for fewer interaction forces between wear and the exoskeleton during different locomotion models. Three years with different heights and varying walking speeds are chosen to test the proposed control strategy and 20 gait iterations are required for parameters regression. The proposed control strategy shows better performance than the traditional Soft Actor–Critic method in handling the inaccurate dynamic model situation and reducing interaction force. Moreover, to overcome the drawback of DMP, trajectory planning methods that focus on the interaction with the environment and neglect the interaction between users and exoskeleton were proposed, Huang et al. [76] presented a Coupled Cooperative Primitive strategy to model motion trajectory online, with an impedance model to monitor the interaction and reinforcement learning to adapt parameters online through pHRI. The experiment shows positive results in tracking users’ motion with reduced pHRI and the ability to adapt to different users. In addition, with regard to personalized parameters of prosthesis control in clinics, compared with traditional labor-intensive ways, Wen et al. [77] proposed an online model-free reinforcement learning control strategy to automatically tune the impedance parameters that meet individual characteristics as needed. For better real-life implementation, the author also considers other situations and adds to the system, for example, the tuning iteration was limited to 70 times to avoid human fatigue in the tuning procedure and restrict the range of robot motion to −5 degrees and 60 degrees to avoid mechanical part collision. The final experiments showed the accuracy of the pre-tuning and post-tuning exoskeleton by evaluating the RMS error and the stability of the proposed tuner was also verified. Rose et al. [96] proposed an end-to-end deep reinforcement learning method to output desired torque values of lower limb joint actuators, which learn by observing joint information. Unlike other reinforcement learning approaches, this model is only learned from the fully-observable environment. So, the reward function is composed of two components: a reward for the deviation between the current and desired joint angles and a penalty for exceeding a maximum or minimum joint angle. Moreover, Kalani et al. [97] implied a Deep Q Network to optimize the time delay in the delayed output feedback controller according to the walking speed of the wearer so that energy consumption is minimized.

## 4. Sensors

**Figure 3:**
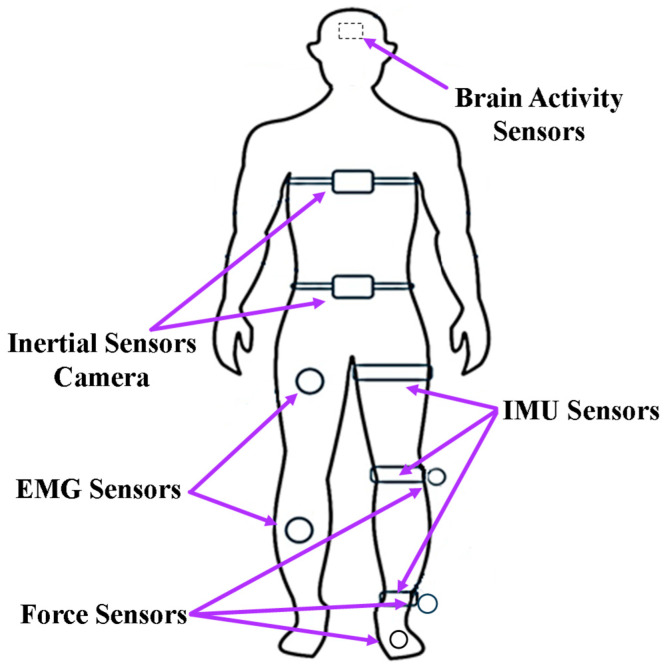
Common attachment locations of sensors.

To realize actively assisted walking, high-performance sensors are essential to provide effective signals for real-time control. Figure 3 shows the most common sensor placement. Based on the controller needed, one or multiple sensors are chosen. Inertial measurement sensors are the most frequently used sensors, such as IMU and accelerometers, which aim to capture the kinematic values and position of the lower limb. Muscle activity sensors, such as EMG and Mechanomyography (MMG), have been rapidly developed because of their advantages in capturing muscle intention. Meanwhile, force sensors are the most commonly used in combination. More and more researchers have adopted the Body Sensor Network (BSN) concept, which aims to fuse multiple sensor signals to assist with complex control commands. Besides those commonly used sensors, brain activity, physiological signals, and computer vision sensors show promise for inclusion in the BSN. The following section summarizes the roles of various sensors in the control algorithm, highlighting their advantages and disadvantages.

### 4.1. Kinematic Sensors

Accelerometers [98], gyroscopes [99], and IMUs [100,101,102,103,104] have been commonly used in activated exoskeletons. These kinematic values, such as instantaneous acceleration and angular velocities, can help determine the angles and segment positions, which are further used for decision-level control such as gait phase detection. In addition to the IMUs, joint angle sensors [105] are commonly used due to their robustness and absolute position information. Moreover, more electronically intelligent sensors such as electronic goniometers have been developed [106], which could record and process the kinematic values of motion and transmit them to the controller directly. While kinematic sensors offer advantages such as non-contact, being cheap, being easy to wear, etc., they also have certain limitations that should be considered. Firstly, they need periodic recalibration to account for signal offset caused by temperature changes, gain fluctuations, and mechanical wear. Additionally, the precision could be challenged due to the vibration and attachment location movement. Moreover, gravity could also affect the values, which need certain compensation at specific axes. In conclusion, kinematic sensors usually require signal processing to deal with drift and erroneous spike problems.

### 4.2. Kinetics Sensors

Kinetics sensors such as strain gauge sensors and piezoelectric sensors are used in exoskeleton devices. Rather than attaching to users’ bodies, they are usually placed on the device itself to collect force, torque, or pressure data for calibrating the execution level control. Foot sensors, such as footswitches and force-sensitive resistors (FSRs), are chosen to provide the ground truth for some motion intention detection [107,108]. Not only could they detect the foot-on and foot-off event but they could also accurately measure the ground reaction force during the gait cycle. However, the foot sensors are prone to mechanical failure [109]. Additionally, proper placement of the sensors could be essential to collect data in the long term stably.

### 4.3. Muscle Activity Sensors

Muscle activity sensors are widely utilized in decision-level control since the electrical signals of muscles are prior to the movements. EMG sensors could capture specific muscle conditions and then the signals could be processed by specific algorithms to link limb motion and neuromuscular activity [110,111,112,113]. Another class of sensors focuses on capturing changes in the mechanical properties of muscles. These include muscle elastography sensors [114], muscle stretch sensors [115], muscle pressure sensors [116,117], and resonance muscle stiffness sensors [118]. Muscle elastography sensors utilize ultrasound techniques to non-invasively assess localized muscle stiffness. Muscle stretch sensors, typically made of conductive plastic material, change their resistance based on the degree of stretch, allowing for the measurement of muscle deformation. Muscle pressure sensors, based on the piezo-resistance principle, measure the pressure exerted by the muscle they are attached to. Additionally, muscle stiffness sensors measure resonance signal changes to determine muscle stiffness. However, these sensors have the disadvantage of slower mechanical sensing of muscle contractions compared to EMG signal measurements [119]. MMG is a measurement technique that records muscle activity based on the vibrations produced during muscle contractions. Various transducers, such as accelerometers, microphones, or laser distance sensors, convert these mechanical vibrations into electrical signals [120,121]. Research has shown that MMG measurements obtained through microphone sensors on different days are reliable and correspond to changes in forces [122]. However, it is important to consider various factors that influence MMG signals, including contact pressure, temperature, and sensor positioning, before employing them in exoskeleton applications.

### 4.4. Brain Activity Sensors

Over the past few years, the utilization of Brain–Computer Interface (BCI) technology for rehabilitating motor disorders has significantly expanded. This therapeutic approach has seen various applications, such as the incorporation of Motor Imagery (MI) and Virtual Reality (VR) in post-stroke therapy [123,124,125]. With the success in upper limb exoskeleton development [126], this technique also shows potential in PPLLE.

Non-invasive brain activity measurements are preferred in the field of the exoskeleton, with an Electroencephalogram (EEG) being a commonly used method [123,127]. EEG involves placing electrodes on the scalp to capture electrical signals from the brain. While EEG provides fine temporal resolution but limited spatial resolution, other non-invasive techniques such as fMRI, fNIRS, MEG, and functional transcranial Doppler ultrasonography have also been considered in BCI [128,129]. EEG signals can be supplemented with Blood-Oxygen-Level-Dependent (BOLD) activity analysis, typically captured by fMRI or fNIRS. Integrating multiple modalities, such as EEG with fNIRS or MEG, can provide complementary information for analyzing brain activity in the context of exoskeletons.

Despite the advantages of non-invasive techniques like EEG, it should be noted that they still have limitations. For instance, EEG requires the use of gel or saline liquid to improve electrode-skin contact, although dry electrodes have been developed as an alternative. On the other hand, fMRI and MEG offer better spatial and temporal resolution but are less suitable for exoskeleton applications due to their size, cost, and lack of portability. In summary, non-invasive techniques like EEG, fNIRS, and their integration hold promise for measuring and analyzing brain activity in the context of exoskeletons, offering potential advancements in the field of robotics.

### 4.5. Computer Vision and Range Sensors

In order to meet the needs of more complex environments, rather than just being confined to the indoor environment, especially in the laboratory, visual and ranging sensors are increasingly being used. For example, real-time images could be captured by wearable cameras and classified into different terrains by extracting features through neural networks [130,131,132,133,134,135]. The most outperformed convolutional neural network achieved 99% classification accuracy for three environment classes, which are level walking, stair ascent, and stair descent [132]. In addition, range sensors could detect environmental properties, such as depth cameras and Lidar [136,137,138]. Compared with neuromuscular-mechanical signals, vision systems can be user-independent, which is more adaptive to different users in complex environments. With the combination of vision cameras and other body capture sensors, the exoskeleton could better locate the limb position and help make further control decisions [139]. However, since it could generate a larger database, signal processing time, synchronism, and battery requirements are big challenges when applied to complex outdoor environments. An open-source large-scale image dataset is needed for researchers to decrease repetitive measurements and make direct comparisons [140].

## 5. Discussion

As shown in Table 6, among the reviewed mechanisms, the flexible PPLLE stands out as a versatile option that combines the advantages of rigidity and softness, making it suitable for various application scenarios. Moreover, its comfort and safety meet the requirements of the users effectively. When larger volumes and masses are allowed, technologies such as springs, cables, and variable stiffness mechanisms can be employed to enhance the performance of exoskeletons. Although the flexible PPLLE exhibits significant potential as a research direction, researchers should also consider exosuits or rigid exoskeletons when evaluating the suitability for other scenarios, such as those involving high force output or precise control capabilities. The selection of the most appropriate exoskeleton robot depends on the specific objectives and application scenarios. We should increase the advantages and mitigate the disadvantages of existing exoskeletons by improving and adding new mechanisms. At the same time, exploring new technologies is also crucial for expanding the application field of exoskeletons and making them more practical.

For instance, the cable-driven PPLLE system requires integration with additional components, typically involving motors, giving it the benefits of motor-driven systems. Moreover, remote cable-driven systems can minimize motion inertia at the end of limbs, but they add complexity to the structure since cables need to be designed in parallel to generate contact force. Furthermore, maintaining appropriate cable tension during operation is essential and can be achieved by incorporating suitable mechanisms. Tensioners, commonly used in transmission systems, offer both manual and automatic solutions to meet different application requirements.

Regarding exosuits, the absence of rigid components in the limbs makes them significantly lighter and contributes to better fit and adaptability to different wearers’ bodies. This characteristic enhances the wearer’s comfort and safety. However, exosuits do not provide supporting forces but rather augment human movement. Consequently, their control may be less precise. One potential solution is to develop a structure such as a gooseneck mechanism [141], which combines flexibility and stiffness to provide adequate support for exosuits. Additionally, customization is crucial as exoskeletons must accommodate individual user needs. This requirement complicates manufacturing, especially for flexible materials, as the process is both time-consuming and labor-intensive. While 3D printing of soft materials is not yet widely commercialized, it holds significant potential to advance exosuit development in the future.

In terms of soft actuators, apart from pneumatic and cable-driven exosuits, promising actuators such as DEA and HSAEL have emerged, requiring hundreds or even thousands of volts to operate. Although advancements in materials, manufacturing techniques, and structures have significantly improved their stability and safety, challenges still exist, especially in the context of HRI. Nevertheless, owing to their high power-to-weight ratio, lightweight and simple structures, and reduced dependence on complex external power systems, these technologies hold great promise for the field of exoskeletons.

In summary, PPLLEs follow two different design philosophies [142]. The first focuses on weight-support systems, ensuring safety for individuals with insufficient lower limb strength during walking or those performing heavy labor tasks, such as repetitive lifting or unloading in factories. This approach is primarily implemented with rigid exoskeletons, which are bulky and energy-intensive, limiting their application to controlled environments like factories, rehabilitation centers, and hospitals. The second design transfers all the weight to the ground, typically using motor-driven cable actuation. While lightweight, these systems lack stability, making them suitable for indoor rehabilitation (with crutch assistance or treadmills) or outdoor activities such as hiking, search-and-rescue missions, or military operations. In contrast, pneumatic and other soft exosuits are currently less industrialized for lower limb applications. Their primary use remains in medical rehabilitation for the upper limbs, particularly in regulated environments.

In terms of the control strategy of exoskeletons, it faces challenges in achieving a universal solution, primarily due to the extensive diversity in application requirements, sensor selection, structural design, and actuator characteristics. The traditional PID controller demonstrates its limitations in achieving accurate trajectory tracking and agile environments adapting, which is primarily attributed to its inherent inability to effectively handle internal interaction noise and external disturbances. Significant advancements have been made in the development of the PPLLE control system, which has addressed many of the previously discussed issues.

Firstly, researchers have directed their efforts toward comprehending and capturing the user’s motion attention to enhance control performance. They employ physical parameters, such as velocity, acceleration, center of mass, etc., and neural signals, like EMG, since they offer a more direct muscle information approach. However, due to sensor precision limitations, human structure variations, and demanding application conditions, the measurement results may not always be entirely reliable. To address this, numerous researchers have begun integrating various components to achieve a more precise estimation of the user’s status. Additionally, the emergence of intelligent control methods, including machine learning techniques and fuzzy theory, has shown superior performance by effectively dealing with noise interference and unmodeled situations. Nevertheless, these methods require more computational resources and time. Although such approaches have shown promise, they are still primarily in the laboratory stage (simulation software or limited DoFs). The real-world performance of these techniques remains unclear.

Secondly, achieving effective human–exoskeleton control is complicated by the varying characteristics of users, such as differences in height, weight, walk habits, and other factors. Maximizing the benefits of exoskeleton assistance requires personalized adjustments, which present a significant challenge outside of a controlled laboratory environment. In previous designs [143], joint angles and torques for the walking cycle were primarily derived from limited resources like Clinical Gait Analysis (CGA) data and experience from medical professionals. To enhance the proposed control system’s generalizability and account for variations in individual gait and different measurement methods, a broader range of independent data sources was utilized. Despite these improvements, the adjustment time to optimize the exoskeletons for different users remains relatively long. For instance, with ReWalk [144], the process of teaching them how to use the exoskeleton and fitting took approximately 30 min per participant. Advanced technology has worked on this; a well-established approach is the utilization of parameters adjustable for a PID-based controller, automatic tuning impedance parameter [145], and experience-based fuzzy rule [146], which relate to the user’s feedback and environment. Alternatively, DL/DRL is an alternative option that can provide optimal and adaptive personalized torque assistance [147] or penalize both interaction force and trajectory modification through the cost function [148]. However, it should be noted that this method requests substantial computational resources. Furthermore, a massive data-driven model can significantly enhance the optimization of control laws [146]. The review by Tao J. et al. [149] also discussed the development and problems of personalized lower limb exoskeleton.

Last but not least, rather than focusing solely on improving tracking accuracy, it is also crucial to pay attention to the human–exoskeleton interaction/coupling, a field that has garnered less attention. This interaction is pivotal in identifying the human’s motion intention, improving control accuracy, and enhancing user comfort, crucial for sustained performance during prolonged use of the exoskeleton. Several studies have focused on this field, based on the modeling interaction as a linear damped springs system [150,151], Neighbourhood Field Optimization (NFO) [152], nonlinear model [153], Coupled Cooperative Primitive (CCP) [76], Sparse Gaussian Process (SGP) [154], backstepping [152,155], Enhanced Whale Optimization Algorithm (EWOA) [156], Particle Swarm Optimization [157], Interaction Predictor [158], and Deep-Gaussian-Process (DGP) [159]. Unlike impedance/admittance control, which relies on predefined virtual coefficients for human–exoskeleton impedance, the human–exoskeleton coupling employs physical parameters to reveal complex dynamics. This approach provides a more accurate representation of the interaction, leading to better performance and user experience. A detailed comparison of different types of control strategies is shown in Table 7.

To achieve the control strategies, multiple sensors are utilized to obtain accurate real-time human movement recognition. IMUs are common, along with force sensors. Muscle activity sensors, such as EMG and MMG, show advantages in motion intent prediction. However, the ease of wearing and durability of use need to be considered, especially for augmentation PPLLE. The increased amount and range of motion pose a challenge for sensors attached to the skin surface. BCI is also a hot-trend solution in prostheses. More researchers choose to apply this technology to upper limb exoskeletons [126,160]. As shown in Table 8, different types of sensors could have different reliabilities and limitations. Considering the complexity of lower limb movements, as well as the synchronization of the user’s limbs and the exoskeleton, how to efficiently apply this technology will be a challenge. Incorporating sensors from different body parts is crucial for identifying challenging movements. Increasing sensor numbers improves accuracy but adds complexity and time delays. An optimal sensor configuration balancing accuracy, complexity, and energy efficiency is achievable, which is essential for exoskeleton design.

The performance of the sensor is highly related to user intent recognition as well as gait analysis, which affects the movement efficiency of the exoskeleton. Consequently, it is better to have a high enough sensor frequency so that the data processing module can capture enough data to identify the features accurately. In addition, it can be found from the previous literature [109,161] that the FSR sensor on the sole of the foot as the ground truth will have the problem of inaccurate and insufficient sensing values. This could cause limitations in the data labeling process. EMG sensors have a similar problem, as the attachment point can change when the user moves, sometimes causing noticeable noise or errors in the data. These low-quality input data could further affect the controllers’ decisions, which leads to a delay or incorrect execution commands that can hinder the user’s movement. Therefore, after the sensor with suitable performance is selected in combination with the data processing unit, the calibration before use and the position deviation during use also need to be carefully managed.

Assessing methods for variability, sensor operability, and daily usage is important. Gait phase detection can utilize various sensor combinations on different body parts, while multiple kinematic sensors enhance movement estimation. Sonomyography (SMG) shows promise for imaging dynamic muscle activity, offering a non-invasive approach with depth sensitivity and minimal interference. SMG’s potential in lower-limb gait analysis requires further investigation. Similarly, fatigue detection in standalone exoskeletons lacks sufficient research. Various sensors such as joint angles, EMG, MMG, SMG, accelerometers, and NIRS hold the potential for assessing fatigue. Exploring these sensors’ capabilities will enhance exoskeleton performance.

Next-generation sensors, such as flexible electronics, dry-electrode EEG systems, and advanced LIDAR, promise significant improvements. Flexible sensors enhance comfort and usability, advanced LIDAR ensures environmental mapping in challenging conditions, and dry-electrode EEG reduces setup time while maintaining signal quality. These innovations make sensors more efficient and adaptable for real-world applications. Ensuring sensor robustness is critical for consistent performance in dynamic environments. Advanced filtering, sensor fusion, and adaptive calibration mitigate noise, drift, and misalignment. For example, fusing data from IMUs, force sensors, and vision systems compensates for individual sensor limitations. Fault-detection algorithms further enhance reliability and safety in real-time operations, crucial for outdoor and extended use.

In the research of PPLLE systems, there are still three important challenges.

(1) The adaptability of the structure

The use of exoskeletons involves a process of putting on and taking off. Exoskeletons with rigid structures connecting joints present challenges in properly fitting the device onto the human body, often requiring significant time and effort. Incorrect placement can result in deviations in the applied force direction and magnitude during operation, leading to inaccuracy from the predetermined control model. Furthermore, the size and space of joints vary from person to person, necessitating customizations for rigid exoskeletons. In contrast, flexible links can mitigate these issues to some extent but introduce complexity in control due to the highly nonlinear nature of the system. The comfort of the fit is also crucial. If it is too tight, it can impact the comfort of the exoskeleton and impede the wearer’s blood circulation. Conversely, if it is too loose, it can result in deviations from the original fixed position after prolonged use.

Regarding safety and comfort, rigid exoskeletons heavily rely on sensors and adaptive algorithms to ensure safety. Although reliability is generally high, the current cumbersome of rigid exoskeletons often compromises wearer comfort. Flexible exoskeletons, on the other hand, significantly improve the safety of interaction and wearing comfort through the inherent flexibility of their materials. However, flexible exoskeletons face challenges in terms of reliability, including shorter lifespans, susceptibility to breakage, fatigue, and performance degradation after prolonged use. Therefore, the choice of materials significantly impacts the overall weight and performance of exoskeleton robots. This is also important for exosuits, which rely entirely on material properties. Due to their limited stiffness, exosuits cannot independently support the body’s weight. Material innovations are essential to enhance mechanical properties, enabling exosuits to provide sufficient support while remaining lightweight and portable. However, these advancements must also address challenges such as nonlinear behavior and stiffness degradation. These issues require urgent attention and resolution.

(2) Intention Prediction

The current control system shows specific shortcomings in predicting the actual intention because it is affected by a large number of physical, biological, and psychological factors and multiple muscle participation. Additionally, owing to the distinct personalities and diverse walking patterns of individuals, the signals indicating the same motion can vary significantly among people. This is extremely common in experiments, which leads to long adjustment processes and inaccurate threshold determination under limited datasets. Furthermore, the complete understanding of how muscles assist human gait remains dimmed. One muscle may exhibit the same behaviors under various locomotion conditions. For example, the gastrocnemius muscle, a large muscle located in the posterior leg, shrinks during the walk and stance phases, respectively. Moreover, certain motions may not necessarily involve the participation of adjacent muscles. An example of this is the cucullate muscle, located in the back, which contributes to the rotation and balance of the torso during walking. These complexities in muscle behavior and involvement make the prediction of a motion challenging. Luckily, computational techniques, especially Artificial Intelligence, are found to be useful in dealing with multiple-dimensional highly heterogeneous problems. Therefore, it is necessary to continuously improve the intention prediction accuracy with new methods in order to ensure the robustness of the control system.

(3) Sensor fusion

With the increasing computing power of processors, more researchers are utilizing data from multiple sensors, which could enhance the detection of user movements and intentions while improving safety through risk detection and prevention. Notably, although physiological signal sensors [162], such as heart rate and blood pressure monitors, are less commonly used in PPLLEs, they offer significant benefits. These sensors provide valuable insights into the user’s fatigue and stress levels, enabling portable exoskeletons to make real-time adjustments that enhance comfort and usability in dynamic environments.

Meanwhile, brain activity sensors and computer vision offer potential advancements in PPLLEs for intention detection and risk detection. BCIs can decode user intentions through neural signals, enabling a more intuitive control mechanism, while computer vision systems provide rich environmental context for adaptive navigation. The combination of these technologies can create a seamless interaction between the user, the exoskeleton, and the environment. However, this fusion introduces challenges, such as high computational demands, real-time data processing, and the need for robust integration algorithms. For example, aligning neural signals from BCIs with visual data from computer vision requires advanced fusion frameworks, such as deep learning-based models or reinforcement learning approaches, to ensure coherent and adaptive decision-making.

Wearability is also key to expanding applications beyond the lab. Lightweight, low-power, and ergonomic designs ensure comfort and practicality in outdoor scenarios. In conclusion, while integrating BCIs, computer vision, and next-generation sensors with PPLLEs holds transformative potential, challenges like synchronization, computational efficiency, robustness, and wearability must be addressed. Developing intelligent algorithms to effectively utilize sensor signals is vital for realizing the full potential of these systems.

## 6. Conclusions

The current trend is to merge emerging technologies, which not only opens up a multitude of possibilities but also poses challenges for decision making. Selecting the appropriate framework and approach in terms of design, control, and sensors is a complex task for researchers. This review provided a comprehensive overview of existing PPLLEs, categorizing each technology by examining a wide range of references. We discussed their advantages, disadvantages, and applicable scenarios in detail, offering insights that can help researchers and developers better understand the latest progress in this field. Despite significant advancements in mechanical design and control strategies, exoskeletons are now applied across diverse domains, including firefighting, medicine, manufacturing, life-saving, and military operations. However, substantial challenges still limit their practicality and widespread adoption, as highlighted in this paper. We also proposed potential solutions and outlined future directions for development to address these challenges. Additionally, in this practically oriented field, integrating new technologies is crucial for addressing diverse application scenarios and accelerating the adoption of exoskeleton robots in everyday life. By promoting rapid technological development driven by commercial interests, this approach aims to make exoskeletons more accessible and valuable in everyday life.

## Figures and Tables

**Table 1:** Technical data of SEA PPLLEs.

	Qian et al. [20,21]	Song et al. [22]	Meijneke et al. [23]
	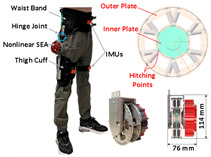	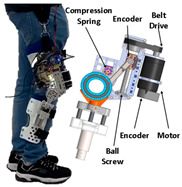	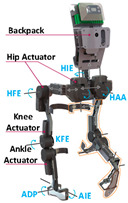
Year	2023	2023	2021
Active Joint	hip	Knee	hip, knee, ankle
Angle (°)	hip (−135~135)	knee (0~120)	-
Speed (m/s)	0.8	1.2	1.3
Continuous Torque (Nm)	6.6	45	-
Max torque (Nm)	19.8	102	102
Stiffness	24.5 KN/m	(220~440) N·m/rad	1500 Nm/rad
Exoskeleton’s Weight (kg)	2 W	1.4 OL W	12.8 + 24.4 W
Load (kg)	-	-	-
	Zhang et al. [24,25]	Zhang et al. [26]	Kang et al. [27]
	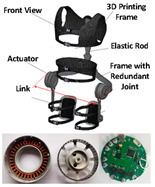	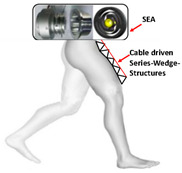	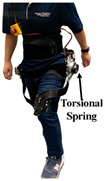
Year	2022	2022	2023
Active Joint	hip	hip	hip
Angle (°)	-	-	hip (−130~130)
Speed (m/s)	1.4	2.2	-
Continuous Torque (Nm)	-	27	60
Max torque (Nm)	62	-	108
Stiffness	(0.7 or 0.9) Nm/deg	-	593 Nm/rad
Exoskeleton’s Weight (kg)	2.95 W	1.4	4.8 W
Load (kg)	4	-	-

OL: only one leg’s weight; W: the weight of or including battery and external power.

**Table 2:** Technical data of VSA PPLLEs.

	Kimura et al. [31]	Shao et al. [32]	Bergmann et al. [33]
	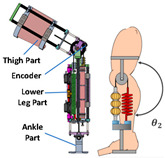	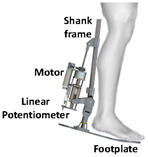	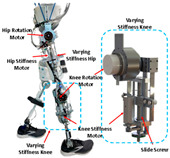
Year	2021	2021	2022
Active Joint	knee	ankle	hip, knee
Angle (°)	knee (0~90)	ankle (−45~135)	-
Speed (m/s)	1.25	-	0.28
Continuous Torque (Nm)	-	-	hip 64.2, knee 40.1
Max torque (Nm)	20	-	-
Stiffness	(0~0.197) Nm/deg	(10~rigid) N/mm	hip (265~515), knee (196~408) Nm/rad
Exoskeleton’s Weight (kg)	3.1 OL	0.77 per actuator	14.4 W
	Sarani et al. [34]	Zhu et al. [35]	Hu et al. [36]
	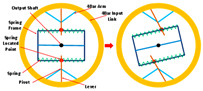	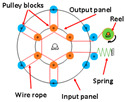	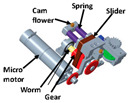
Year	2022	2022	2023
Active Joint	knee	knee	hip
Angle (°)	knee (−180~180)	knee (0~135)	hip (−20~20)
Speed (m/s)	-	-	-
Continuous Torque (Nm)	99.76	19	17.5~49
Max torque (Nm)	-	66.6	-
Stiffness	(98~533.6) Nm/rad	(3.5~549) Nm/rad	(50~120) Nm/rad
Exoskeleton’s Weight (kg)	3 per actuator	2.8 OL W	3.32

OL: only one leg’s weight; W: the weight of or including battery and external power.

**Table 3:** Technical data of PPLLEC.

	Chan et al. [38]	Park et al. [39]	Kieuvongngam et al. [40]
	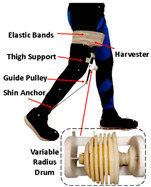	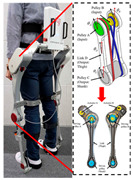	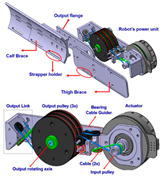
Year	2021	2021	2022
Active Joint	knee	hip, knee	knee
Angle (°)	-	-	knee (0~70)
Speed (m/s)	1.11	1.22	-
Continuous Torque (Nm)	-	-	-
Max torque (Nm)	20	hip 62, knee 78.12	-
Exoskeleton’s Weight (kg)	0.483 OL	14 W	2.1 W
	Orekhov et al. [41]	Chen et al. [42]	Zhong et al. [43]
	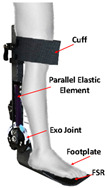	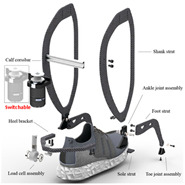	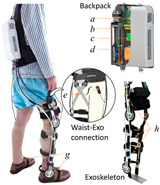
Year	2022	2022	2023
Active Joint	ankle	ankle	hip, knee, ankle
Angle (°)	-	ankle (−50~30)	-
Speed (m/s)	0.75~1.25	1.25 or 2	-
Continuous Torque (Nm)	19.9	-	-
Max torque (Nm)	22	50	17
Exoskeleton’s Weight (kg)	2.5 W	2.55 W	4.5 W

OL: only one leg’s weight; W: the weight of or including battery and external power.

**Table 4:** Technical data of PPLLEC-EXOSUIT.

	Wang et al. [46]	Ye et al. [47]	Ma et al. [48]	Lee et al. [49,50]
	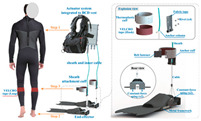	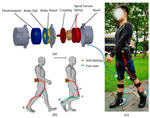	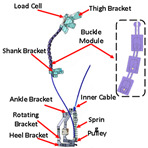	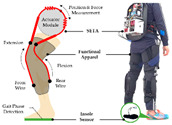
Year	2020	2021	2022	2022
Active Joint	ankle	hip, ankle	knee, ankle	knee
Speed (m/s)	-	-	1.25	1.11
Continuous Torque or Force	840 N	-	-	38 N
Max Torque or Force	1700 N	hip 62.5 N, ankle 80 N	600 N	226 N
Exoskeleton’s Weight (kg)	2.9 W	2.24 W	5.4 W	6.9 W
	Biao et al. [51]	Wang et al. [52]	Wu et al. [53]	Xu et al. [54]
	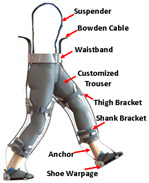	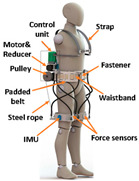	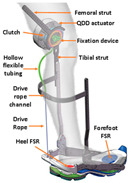	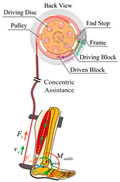
Year	2023	2024	2024	2024
Active Joint	hip, knee, ankle	hip	knee, ankle	ankle
Speed (m/s)	0.25	0.833	0.78~1.48	-
Continuous Torque or Force	-	-	18	8
Max Torque or Force	hip 32 Nm, knee 21 Nm, ankle 13 Nm	32.62	23	16
Exoskeleton’s Weight (kg)	4.8 W	3.62 W	1.4 OL + 1.1 W	1.1 OL

OL: only one leg’s weight; W: the weight of or including battery and external power.

**Table 5:** Technical data of PPLLEP-EXOSUITs.

	Thalman et al. [55]	Veale et al. [56]	Park et al. [57]	Miller-Jackson et al. [58]	Yilmaz et al. [59]
	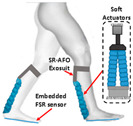	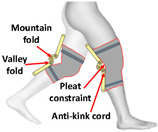	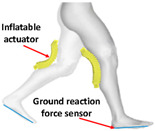	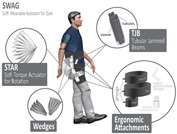	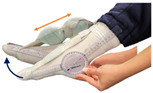
Year	2020	2020	2020	2022	2024
Active Joint	ankle	knee	knee	hip	ankle
Angle (°)	ankle (−20~30)	knee (0~82)	knee (0~160)	hip (−15~30)	ankle (0~20)
Max Torque or Force	118.2 ± 3.1 N	324 Nm	12.3 Nm	31 Nm	107
Exoskeleton’s Weight (kg)	-	1.95 OL	0.1 OL	-	-

OL: only one leg’s weight; W: the weight of or including battery and external power.

**Table 6:** Summary of different types of PPLLE.

Flexibility	Types	Benefits	Drawbacks
Rigid	Hydraulic actuator	Stable and high driving force;	Common: Bulky, uncomfortable, high inertia and structural complexity; Hard to tightly fit to the wearer might cause misalignment issues; Hydraulic: Low driving speed; High pollution due to oil leakage; Motor: Low stability impact;
	Motor actuator	Stable, driving force, fast response, compact and high control accuracy; Rotation range is only limited by rigid fixed structure;	
Flexible	SEA and RSEA	High fidelity torque control, enhances the safety and robustness of the structure; Lower mechanical impedance and shock tolerance;	Bulky and complex structure;
	Variable stiffness	Variable stiffness can ensure the wearer’s safety, improve shock absorption performance, and reduce control complexity and energy consumption;	Bulky complex structure and lack of robustness;
	Cable connection	The small motion inertia of the limb ends, and the simple structure can greatly enhance human interaction safety and minimize the wearer’s discomfort;	The Bowden cable may experience intermittent instantaneous relaxation;
Soft	Cable exosuit	Rapid response, lightweight and simple structure; Can fit wearer’s body;	The Bowden cable may experience intermittent instantaneous relaxation;
	Pneumatic exosuit	Common: Lightweight, safe, high force/weight ratio; Friendly interaction with humans; Comfortable and can fit wearer’s body; Pneumatic: Safer; PVC: Low cost; SMA: Simple structure; DEA: High response speed;	Common: Low stiffness, hard to control precisely, cannot support the wearer because of low stiffness; Pneumatic: Poor air tightness, air pumps are required, noise from the air pumps; PVC: Performance will decline with long-term use, not environmentally friendly; SMA: Slow response with a nonlinear hysteresis effect; High temperatures may be harmful to the human body; DEA: High driving voltage, which may harm humans;
	Others		

**Table 7:** Comparison of different types of control strategies.

Level	Strategy	Principle	Advantages
Decision level	Rule-based	Use predefined logic based on experience to govern the movements and responses	- The deterministic nature of rule-based systems makes them straight forward to design and implement - Lack of complex algorithms which reduced computational requirements
	Pre-trained	Based on prior data to capturing user behavior	- Handle diverse user behaviors which are suitable for wide range of task - Robust to data contamination
	Model-free	Learn to control through real-time interaction and feedback	- Performs well in unpredictable or highly dynamic environments - Adapt to different users or tasks without requiring significant manual reconfiguration
Execution level	Impedance control	Create virtual mechanical impedance (mass, damping, and stiffness) between the exoskeleton and user	- Ensure compliant and smooth interactions, reducing the risk of injury - Adjust to suit different users, tasks or environmental conditions by modifying the impedance parameters
	Predictive control	Use dynamic model of the system to predict and optimize the behavior over a future time horizon	- Optimal control by considering system dynamics and objectives over a prediction horizon - Explicitly incorporates physical and operational constraints, ensuring safe and feasible control
	EMG-based control	Utilizes electrical signals generated by muscle activity to govern the exoskeleton	- Provide an intuitive and seamless interface directly from muscle activity - Unique to everyone, allowing for highly personalized control strategies
	Fuzzy control	Use fuzzy logic to handle systems with uncertainty, imprecision, or nonlinearity	- Effectively manages imprecise or noisy inputs, robust in real-world scenarios - No requirement for an exact mathematical model of the system
	Reinforcement learning/Deep reinforcement learning	The exoskeleton learn to make decisions by interacting with an pre-defined environment	- Highly adaptable to dynamic environments and task since it learns directly from interaction - No requirement of an explicit model, suitable for complex systems

**Table 8:** Comparison of different types of sensors.

Sensors	Reliability	Limitations
Kinematic Sensors	- Provide detailed motion data (e.g., accelerations, angular velocities) with high temporal resolution. - Useful for real-time gait phase detection and activity monitoring.	- Susceptible to drift and noise over extended use, especially in dynamic movements. - Require robust calibration and placement for accurate readings.
Kinetics Sensors	- Deliver accurate measurements of ground reaction forces and pressure distribution. - Essential for understanding load dynamics during ambulation.	- Often bulky and difficult to integrate into wearable systems. - Sensitive to surface irregularities and misalignment.
Muscle Activity Sensors	- Provide direct insight into muscle activation patterns, essential for user intent detection. - Widely validated in laboratory conditions.	- Signal quality is highly affected by motion artifacts, skin impedance, and electrode placement. - Limited ability to capture deeper muscle activities or consistent readings during prolonged use.
Brain Activity Sensors	- Allow for intuitive user intent detection by directly interpreting neural signals. - Effective in controlled environments and low-noise conditions.	- Prone to interference from electrical noise and environmental factors. - Long setup times and potential discomfort with traditional wet electrodes. - Dry electrode systems may sacrifice signal quality.
Computer Vision and Range Sensors	- Excellent for environmental awareness, obstacle detection, and terrain mapping. - Capable of capturing 3D spatial information for navigation and adjustment.	- Performance degrades in low-light conditions or with reflective surfaces. - High computational demands and integration complexity in wearable systems.

## Data Availability

No new data were created or analyzed in this study.
